# The Maternal–Fetal Interface in Small-for-Gestational-Age Pregnancies Is Associated With a Reduced Quantity of Human Decidual NK Cells With Weaker Functional Ability

**DOI:** 10.3389/fcell.2020.00633

**Published:** 2020-09-03

**Authors:** Fang Lin, Chuan Yang, Ting Feng, Shuo Yang, Rong Zhou, Hong Li

**Affiliations:** ^1^Center for Translational Medicine, Key Laboratory of Birth Defects and Related Diseases of Women and Children (Sichuan University), Ministry of Education, West China Second University Hospital, Sichuan University, Chengdu, China; ^2^Department of Obstetrics and Gynecology, West China Second University Hospital, Sichuan University, Chengdu, China

**Keywords:** small for gestational age, maternal-fetal interface, decidua, natural killer cells, innate immunity

## Abstract

Small for gestational age (SGA) refers to a birth weight that is less than the 10th percentile of the mean weight of infants at the same gestational age. This condition is associated with a variety of complications, and a high risk of cardiovascular and cerebrovascular diseases in adulthood. Decidual natural killer (dNK) cells at the maternal–fetal interface have received significant research attention in terms of normal pregnancy or miscarriage; however, data relating to SGA are limited. In this study, we aimed to investigate the characteristics and regulatory role of dNK cells at the maternal–fetal interface in SGA. Using immunofluorescence assays, we found that dNK cells maintained close contact with extra-villous trophoblasts, and the proportion of dNK cells in SGA decreased more than in appropriate for gestational age (AGA). Flow cytometry also showed that there was a significantly lower percentage of dNK cells in SGA (25.01 ± 2.43%) than in AGA (34.25 ± 2.30%) (*p* = 0.0103). The expression of the inhibitory receptor NKG2A on dNK cells and the secretion levels of both perforin and TGF-β1 from dNK cells were significantly higher in SGA than in AGA, while the cytotoxicity of dNK cells in SGA against K562 cells was attenuated. Compared to AGA, the functional ability of dNK cells in SGA showed significant functional impairment in promoting proliferation, migration, invasion, and tube formation in trophoblast cells or vascular endothelial cells. The abnormal function of dNK cells may affect fetal growth and development, and could therefore participate in the pathogenesis of SGA.

## Introduction

Small for gestational age (SGA) refers to a condition in which the birth weight is less than the 10th percentile of the mean weight of infants at the same gestational age (Iams, 2010). SGA is one of the most dangerous conditions for neonates as it is often associated with dysplasia and other serious complications, such as asphyxia and infection (Chan et al., 2010; Li et al., 2013). SGA is also related to an increased risk of other diseases in adulthood, including hypertension, coronary disease, stroke, diabetes, and chronic renal impairment (Bamfo et al., 2007; Hogeveen et al., 2012; Strambi et al., 2012; Mericq et al., 2016; Vollsaeter et al., 2018). The descendants of SGA adults are also associated with a high risk of subsequent SGA, or any form of placental-mediated disease (Sepúlveda-Martínez et al., 2018). Due to the increasing numbers of pregnancies in women of advanced age, and the increased application of assisted reproductive technology (ART), the incidence of SGA has reached 8.77% in the mainland of China (Liu et al., 2014). Consequently, the pathogenesis of SGA, and the development of new treatment strategies, has become an urgent research issue in the field of pediatrics and perinatal medicine.

The maternal–fetal interface is the direct point of contact between the mother and the fetus. The immune microenvironment at the maternal–fetal interface is essential for a normal pregnancy, and features the participation of trophoblast cells, a variety of immune cells (NK cells, T lymphocytes, macrophages, and dendritic cells), and cytokines (Sullivan et al., 2008; Arck and Hecher, 2013). Decidual NK (dNK) cells are known to be the most predominant form of lymphocytes at the maternal–fetal interface during the first trimester, and exhibit a unique range of phenotypic and functional characteristics (Moffett-King, 2002; Manaster and Mandelboim, 2008). Approximately 90% of dNK cells are CD56^bright^CD16^–^, whereas the major subset of NK cells in peripheral blood are CD56^dim^CD16^+^ and associated with cytotoxic activity (Koopman et al., 2003). In a healthy human pregnancy, dNK cells exhibit poor cytotoxicity, can regulate local immune balance, and promote trophoblast invasion and spiral artery remodeling (Kalkunte et al., 2009; Vacca et al., 2011), which is conducive to the maintenance of pregnancy and the growth and development of the fetus. Studies focused more on dNK cells in early pregnancy but less in late gestation. According to previous studies, dNK cells in late gestation had the same phenotype to those found during early pregnancy (Sánchez-Rodríguez et al., 2011). The status of NK cells changed dynamically with the development of the pregnancy. The number of dNK cells did not alter between 1st and 2nd trimester pregnancy, while a characteristic decline in the percentage of CD56^bright^CD16^–^ dNK cells occurs in late gestation (Sindram-Trujillo et al., 2003; Williams et al., 2009b). The persistence of this subset until the end of pregnancy suggests that they may be of physiological relevance throughout pregnancy (Sánchez-Rodríguez et al., 2011).

Numerous studies have investigated immune cells at the maternal–fetal interface; however, the immune microenvironment of SGA has yet to be reported. In a previous paper, we showed that there was a reduction in the number of NK cells in the cord blood of SGA infants and that these cells had a lower antiviral capacity (Li et al., 2014). With the full authorization of China’s second child policy, the risk of SGA will inevitably increase further. Consequently, it is vital that we determine whether abnormalities in the immune microenvironment at the maternal–fetal interface are involved in the pathogenesis of SGA. To identify this hypothesis, we analyzed the specific characteristics of dNK cells, and their regulatory effects on trophoblasts and vascular endothelial cells in SGA.

## Materials and Methods

### Tissue Collection and Ethical Permission

Human decidual tissues were collected from the Department of Obstetrics and Gynecology of the West China Second University Hospital, Sichuan University, between October 2016 and January 2019. Women with endocrine and metabolic diseases (diabetes, hyperthyroidism, and hypothyroidism), hypertension, or infectious diseases (hepatitis, AIDS, syphilis, or any other bacterial or viral infection) were excluded. We also excluded women who had received organ transplantation, immunotherapy, or blood transfusion. Fresh decidual tissues were collected, under aseptic conditions, as soon as possible after cesarean section, and delivered to our laboratory for analysis. The research protocol was approved by the Ethics Committee of West China Second University Hospital of Sichuan University and informed consent was obtained from all participants [Approval number: Medical research 2017 (019)]. All the experiments that involved human specimens were performed in a biosafety cabinet and the specimens were treated according to the guideline of medical waste of the West China Second University Hospital, Sichuan University.

### Hematoxylin and Eosin Staining, and the Immunofluorescence Analysis of Paraffin-Embedded Tissues

Formalin-fixed paraffin-embedded sections were stained with hematoxylin and eosin (H&E) according to standard methods (Feldman and Wolfe, 2014). For immunofluorescence assays, sections were first deparaffinized with xylene and ethanol. We then carried out an antigen-retrieval protocol on the sections and the antigens by incubating in ethylenediaminetetraacetic acid (EDTA) antigen-retrieval buffer in a microwave oven (medium heat for 8 min, power turned off for 8 min, and then low heat for 7 min). Non-specific binding sites were then blocked by incubating the sections in 3% bovine serum albumen (BSA) for 30 min at room temperature. The blocking serum was then removed and the tissue sections were incubated overnight at 4°C with 1:100 diluted rabbit anti-human neural cell adhesion molecule 1 (NCAM1) antibody (Abcam, United States). The following morning, the sections were washed three times and then incubated with a 1:400 diluted horseradish peroxidase (HRP) binding goat anti-rabbit secondary antibody (HuaBio, United States) for 50 min. The sections were washed three times and incubated with CY3-tyramide signal amplification (TSA) (Google Biotechnology, Wuhan) at room temperature in darkness for 10 min. After washing the sections three times, we carried out a microwave treatment to remove the primary antibody and secondary antibody that have been combined to the tissue. Using the same protocol, sections were then re-incubated with 1:100 diluted rabbit anti-human Cytokeratin 8 (Abcam, United States), and a 1:400 diluted HRP binding goat anti-rabbit secondary antibody. FITC-TSA (Google Biotechnology, Wuhan) was added and treated with a microwave. The cell nuclei were stained with 4’,6-diamidino-2-phenylindole (DAPI) (Sigma, United States) for 10 min at room temperature. The final sections were analyzed on an inverted fluorescence microscope (Nikon, Japan; × 200) and quantified by ImageJ (National Institutes of Health, United States) (*n* = 3).

### The Isolation of Decidual Immunocytes

Decidual immunocytes (DICs) were isolated using a previous methodology with some modifications (Fan et al., 2011; Tilburgs et al., 2015). Decidual tissues were separated carefully from the villi, minced into 1–3 mm fragments, and then rinsed in phosphate buffer solution (PBS) containing 0.1% penicillin and streptomycin (Life Technologies, Belgium). Tissues were then digested with 1 mg/ml Collagenase IV (Sigma, United States), and 150 U/ml of DNase I (Applichem, Germany) for 1 h at 37°C with gentle agitation. Digestion was terminated with 10% fetal calf serum (Hyclone, United States). Thereafter, the suspension was successively filtered through 70 and 40 μm pores, centrifuged at 500 × *g* for 10 min at room temperature, and resuspended in 3 ml of complete medium (RPMI 1640 + 10%FBS + 1% penicillin and streptomycin). The final suspension was layered over a pre-formed Percoll gradient (Pharmacia, Sweden) that had been prepared in PBS. We then prepared a 60 to 20% Percoll (vol/vol) gradient by diluting 3 ml fractions of 90% Percoll with PBS. The gradient was then centrifuged at 600 × *g* for 20 min. The 40–60% layer (containing DICs at a density of 1.056 to 1.077 g/ml) was then removed and washed twice with PBS.

### Flow Cytometry

For flow cytometry, mononuclear cells were first resuspended in PBS at a density of 3 × 10^6^/ml. We then added 100 μl aliquots of samples to individual Falcon 2054 polystyrene round-bottom tubes (Becton Dickinson, United States) for immunolabeling. Cells were immediately stained with a range of monoclonal antibodies (BV510-anti-CD45, APC/CY7-anti-CD3, APC-anti-CD56, Percp/cy5.5-anti-CD16, FITC-anti-CD69, and PE-anti-NKG2A (all from BioLegend, United States), PE/CY7-anti-NKG2D, PE-Vio770-anti-KIR2DL1/CD158a (all from Miltenyi, Germany) and PE-anti-KIR2DS1/CD158h (R&D, United States). Following a 30-min incubation at room temperature, the cells were washed twice, and then resuspended in PBS for surface marker analysis by flow cytometry (SGA: *n* = 7–14, AGA: *n* = 12–15). For intracellular expression analysis, isolated cells (5 × 10^5^/ml) were co-cultured with phorbol myristate acetate (PMA; Sigma, United States), ionomycin (Sigma, United States) and brefeldin A (BioLegend, United States) for 4 h in Falcon 2054 polystyrene round-bottom tubes. Thereafter, the cells were harvested and labeled for CD45, CD3, and CD56, then fixed, permeabilized, and stained for Perforin, IFN-γ, IL-17A, TGF-β1, IL-4, IL-8 (all from BioLegend, United States), IL-10, and IP-10 (from BD, United States). Next, the cells were washed and resuspended in PBS for flow cytometry analysis (SGA: *n* = 10, AGA: *n* = 13).

For dNK cell cytotoxicity analysis, we quantified dead cells using a commercial LIVE/DEAD^TM^ Cell-Mediated Cytotoxicity Kit (Invitrogen, United States), in accordance with the manufacturer’s guidelines. Then, 1 × 10^6^ of target cells (K562) were incubated with 10 μL DiOC stock solution (component A) for 20 min at 37°C. The cells were then washed twice with PBS and resuspended in complete culture medium at a concentration of 1 × 10^6^ cells/ml. The effector cells (dNK) and target cells were then mixed at a ratio of 20:1 (E: T = 20:1), and counterstained with diluted propidium iodide with gentle mixing. Next, the cells were pelleted by centrifugation at 1000 × *g* for 30 s and incubated at 37°C for 2 h. After the incubation period, the tubes were tapped gently to dislodge the pellets, resuspended by vortexing, and immediately analyzed by flow cytometry. Dead K562 cells (DiOC^+^ PI^+^ cells) were represented as cytotoxic signals in the upper right-hand quadrant (SGA: *n* = 5, AGA: *n* = 9).

### The Isolation of Decidual NK Cells

NK cells were enriched from the isolated decidual mononuclear cells using a magnetic isolation kit (positive selection) (Miltenyi Biotec, Germany). In brief, the isolated decidual mononuclear cells were suspended in 80 μl of PBS buffer containing 0.5% BSA, 2 mM EDTA, and 20 μl CD56 microbeads, mixed well, and incubated at 4°C for 15 min. The cells were then washed, resuspended in buffer, and passed through a magnetic separation [MS] column (Miltenyi Biotec, Germany). The magnetically labeled NK cells were retained on the column. Next, the column was removed from the magnetic field, and the magnetically retained NK cells were eluted as the positively selected cell fraction. The purity of the enriched NK cells was then evaluated by flow cytometry (Becton Dickinson, United States). The purity of the isolated cells exceeded 90%.

### Proliferation Assays

First, we seeded 1 × 10^5^ trophoblast cell line HTR8/SVneo cells, cultured in RPMI 1640 medium supplemented with 10% fetal bovine serum (FBS), 1% penicillin, and streptomycin in a 24-well plate for adherent culture. Thereafter, 1 × 10^5^ isolated dNK cells were directly co-cultured with HTR8/SVneo cells for 24 h. Then, the dNK cells were discarded, while the HTR8/SVneo cells were washed twice, collected, fixed, permeabilized, and stained with Ki-67. Next, the cells were washed and resuspended in PBS and 7-AAD was added for DNA staining before flow cytometry analysis. The proliferation rate was expressed as the proportion (%) of 7-AAD^+^ Ki-67^+^ S/G2-M cells (*n* = 5).

### Transwell Invasion and Migration Assays

In brief, 8 μm transwell inserts, containing membranes (Corning, United States), were coated with a 5× dilution of Matrigel (Corning, United States) and placed in a 24-well plate. Next, 5 × 10^4^ HTR8/SVneo cells, or 3 × 10^4^ human umbilical vein endothelial cells (HUVECs), resuspended in 500 μl RMPI1640 or EGM-2 medium, were seeded in the upper chamber, while the same number of isolated dNK cells, in 200 μl RMPI1640 or EGM-2 medium supplemented with 10% FBS, were placed in the lower chamber. Following incubation for 24 h, the cells were fixed and stained with crystal violet. Non-invaded cells on the upper surface of the membrane were removed using a cotton swab. The number of stained cells on the bottom surface of the membrane was then determined by light microscopy at a magnification of × 200. All experiments were performed in duplicates, and the invasion index was expressed as the proportion (%) of invaded cells compared with the corresponding control (cultured HTR8/SVneo or HUVECs without dNK cells). The protocol for transwell migration assay was the same as for the invasion assay, except for the addition of Matrigel (*n* = 6).

### Tube Formation Assays

Cellular angiogenesis was evaluated by performing tube formation assays on an extracellular Matrigel; 96-well plates were coated with 60 μl of cold liquid Matrigel (Corning, United States) per well, and then incubated at 37° for 30 min to promote solidification. HTR8/SVneo cells cultured with RPMI 1640 supplemented with 10% FBS, or HUVECs cultured with endothelial cell growth medium-2 (EGM-2), were grown for 24 h to attach to the bottom of the 24-well plate. Afterward, isolated dNK cells were seeded in the 0.4 μm upper chamber and co-cultured with HTR8/SVneo cells or HUVECs which were seeded in the lower chamber for another 24 h. The HTR8/SVneo cells, or HUVECs, were then harvested and seeded to the Matrigel at a density of 2 × 10^4^/well in 50 μl of medium. After 4 h, the wells were analyzed on an inverted microscope (Olympus, Japan). We then quantified the number of nodes, the total branch length, and the total tube length; these data were expressed as pixels and were analyzed by ImageJ (*n* = 6).

### Statistical Analysis

Data are given as mean ± standard deviation (SD) or median according to normality test. Statistical analysis was carried out using unpaired *t*-tests or non-parametric tests, and differences were considered significant when *p* < 0.05. All statistical analyses were performed using GraphPad Prism version 7.0c (GraphPad Software, United States).

## Results

### Clinical Samples

Decidual tissues, including parietalis and basalis, were collected from 48 appropriate for gestational age neonates (AGA; newborn birth weight ranged from 10 to 90% of the average birth weight at the same gestational age) and 24 SGA. All neonates were delivered by elective cesarean section for the convenience and consistency of sample collection. Gestational age, birth weight, birth height, placenta weight, and 1-min Apgar scores were all significantly lower in the SGA group than in the AGA group (Table 1). This indicated that SGA is associated with poorer intrauterine development, and that SGA babies need further medical attention and special care after birth.

**TABLE 1:** Demographic data of AGA and SGA groups.

	AGA (*n* = 48)	SGA (*n* = 24)	*p* value
Maternal Age (years)	30.19 ± 0.55	29.86 ± 1.14	0.76
Gestational Age (weeks)	39(37–41.6)	37.35(29.7–39)	<0.0001
Birth Weight (g)	3319 ± 59.43	2123 ± 95.17	<0.0001
Birth Height (cm)	50(47–53)	46(35–48)	<0.0001
Placenta Weight (g)	510(405–815)	350(300–500)	<0.0001
preterm/full-term	0/48	8/16	ND
infant gender (M/F)	25/23	14/10	ND
Gravidity	2(1–5)	2(1–4)	0.73
Parity	1 ± 0.17	1.5 ± 0.27	0.12
Apgar Score			
1 min	10(9–10)	10(5–10)	0.0004
5 min	10(9–10)	10(9–10)	0.0686
10 min	10(9–10)	10(9–10)	0.0686

*AGA, appropriate for gestational age; SGA, small for gestational age. Numbers are mean ± SD, or median (extremum). ND, not done.*

### Distribution and Phenotype of dNK Cells at the Human Maternal–Fetal Interface in SGA

**FIGURE 1:**
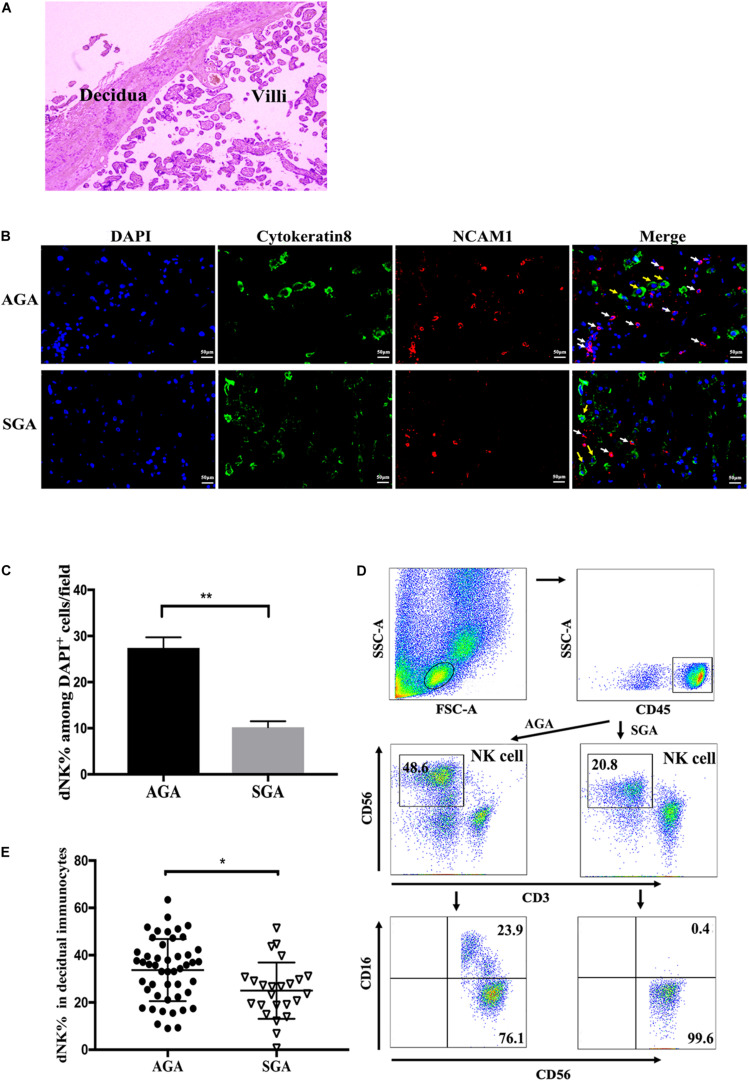
Distribution and phenotypes of dNK cells at the maternal-fetal interface. Paraffin sections of AGA and SGA decidua were stained with NCAM1 (NK cells, red), Cytokeratin 8 (trophoblasts, green), and DAPI (nucleus, blue), and visualized by an inverted fluorescence microscope. **(A)** The location of decidua and villi in the placenta were demonstrated by HE staining. **(B)** The distribution of dNK cells (white arrow) and EVT cells (yellow arrow) in AGA and SGA decidua, as shown by immunofluorescence assay. Scale bars represent 50 μm. **(C)** Percentage of NK cells in all DAPI^+^ nuclei in AGA and SGA decidual tissues in each field, as quantified by ImageJ (*n* = 3). **p < 0.005. **(D)** Representative flow cytometry analysis of the expression of CD3^–^ CD56^+^ decidual NK cells (in the box above) on gated CD45^+^ leukocytes. **(E)** The frequency of dNK cells in SGA (*n* = 24) and AGA (*n* = 48). *p < 0.05.

HE staining and immunofluorescence assays were used to confirm the location of the decidua and villi (Figure 1A), and to demonstrate the distribution of dNK cells and extra villous trophoblast cells (EVTs) in both AGA and SGA decidua (Figure 1B). dNK cells were positive for NCAM1. Cytokeratin 8 staining was used to identify trophoblast cells. The percentage of dNK cells among all DAPI stained nuclei in each field was statistically analyzed by counting the cells in five random fields (at a magnification of × 200). dNK cells and EVTs were found to be adjacent to each other in the decidua (Figure 1B). The percentage of dNK cells in SGA decidua (10.2 ± 0.58%) was significantly lower than that in AGA decidua (27.4 ± 1.03%) (Figure 1C). We then compared the proportion of CD3^–^CD56^+^ dNK cells between SGA and AGA by flow cytometry. After gating lymphocytes and CD45^+^ populations, the proportion of dNK cells became apparent (Figure 1D). The proportion of dNK cells in the SGA group (25.01 ± 2.43%) was significantly lower compared with the AGA group (34.25 ± 2.30%) (Figure 1E).

### The Inhibitory Receptor NKG2A Was Expressed at High Levels on dNK Cells in SGA

**FIGURE 2:**
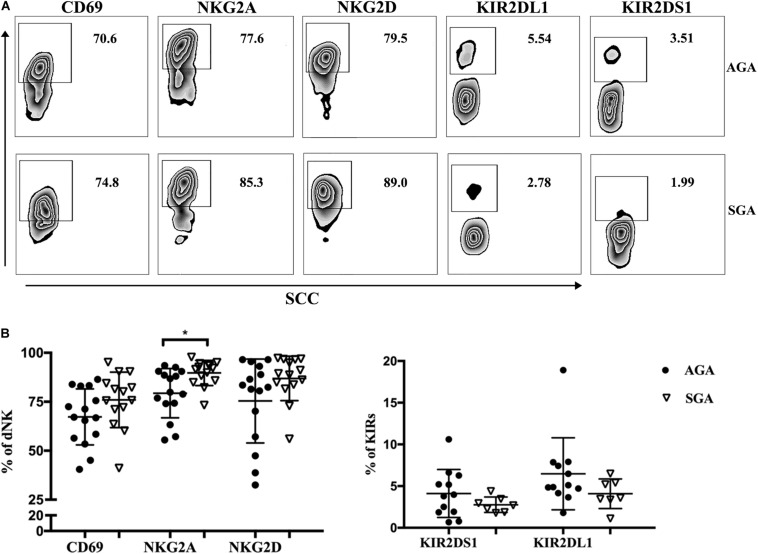
The receptors expressed on dNK cells in SGA. **(A)** Representative flow cytometry analysis of the expression of CD69, NKG2A, NKG2D, KIR2DS1, and KIR2DL1 on gated CD3^–^ CD56^+^ dNK cells in SGA (the lower row) and AGA (the upper row). **(B)** The frequency of CD69, NKG2A, NKG2D, KIR2DS1, and KIR2DL1 on dNK cells in SGA (*n* = 7–14) and AGA (*n* = 12–15). *p < 0.05.

The function of NK cells depends on the expression of activated receptors and inhibitory receptors. We analyzed the expression of several membrane receptors (CD69, NKG2A, NKG2D, KIR2DS1, and KIR2DL1) on dNK cells (Figure 2A). CD69 is a marker of the early activation stage and serves as a co-stimulatory signal to further enhance cell activation, proliferation, and differentiation. NKG2A and KIR2DL1 are inhibitory receptors that transmit inhibitory signals, while NKG2D and KIR2DS1 activate receptors and transmit activated signals. We found that the inhibitory receptor NKG2A was expressed at significantly higher levels on dNK cells in SGA (89.71 ± 1.71%) than in AGA (79.37 ± 3.25%) (Figure 2B). There were no significant differences in the expression of CD69, NKG2D, KIR2DL1, or KIR2DS1 on dNK cells when compared between SGA and AGA. This may suggest that NK cells at the maternal–fetal interface may be relatively immunosuppressive in SGA.

### dNK Cells in the SGA Group Exhibited Lower Levels of Cytotoxicity

**FIGURE 3:**
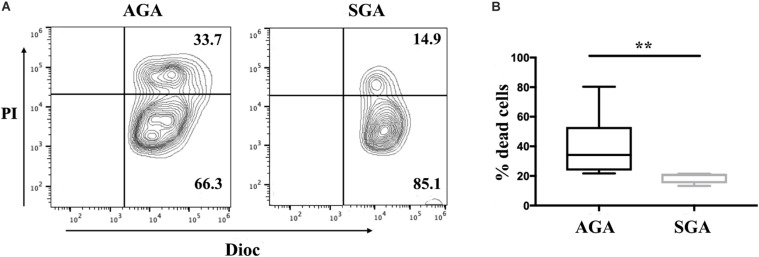
The reduced cytotoxicity of dNK cells in SGA. dNK cells were first incubated with IL-15 (20 ng/ml) for 24 h. Flow cytometry analysis showed the percentage of Dioc^+^ PI^+^ dead K562 cells after gating of Dioc^+^ cells. **(A)** The cytotoxicity of dNK cells from SGA and AGA against K562. Data show a representative experiment in a single individual from each group. **(B)** Statistical analysis of the cytotoxicity of dNK cells in SGA (*n* = 5) and AGA (*n* = 9). **p < 0.005.

Cytotoxicity is a major function of NK cells and also an important indicator of the functional status of NK cells. Here, we used flow cytometry to explore the cytotoxicity of dNK cells against K562 in SGA and AGA when E:T was 20:1. We observed that the cytotoxicity of dNK cells in SGA (19.08 ± 3.88%) was significantly lower than that in AGA (40.44 ± 19.88%) (*p* = 0.0028) (Figure 3). This indicated that the cytotoxicity of dNK cells in the SGA group was significantly reduced during late gestation.

### dNK Cells in the SGA Group Showed a Different Profile of Intracellular Expression

**FIGURE 4:**
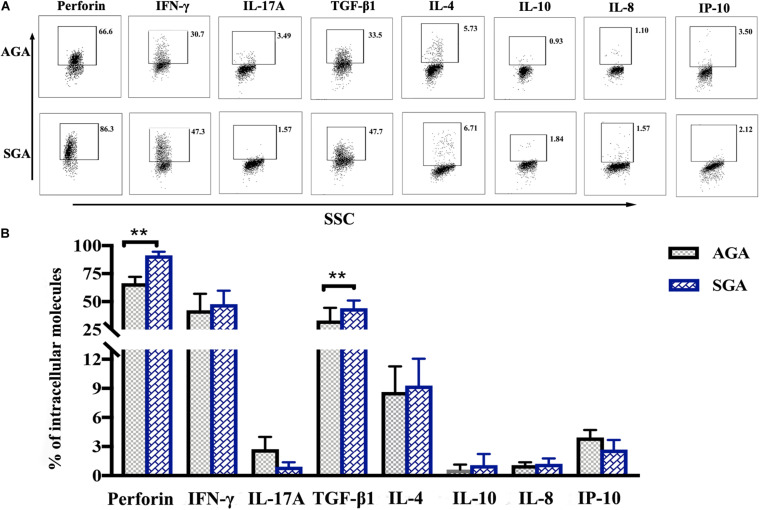
The profile of intracellular expression of dNK cells. Cells were treated with PMA, ionomycin, and brefeldin for 4 h and were then fixed and permeabilized after the surface staining of CD3 and CD56. Cells were then stained for perforin, IFN-γ, IL-17A, TGF-β1, IL-4, IL-10, IL-8, and IP-10. **(A)** Representative flow cytometry analysis of the expression of perforin, IFN-γ, IL-17A, TGF-β1, IL-4, IL-10, IL-8, and IP-10. **(B)** Statistical analysis of intracellular molecules expressed by dNK cells from SGA (*n* = 10) and AGA (*n* = 13). **p < 0.005.

dNK cells appear to have an intrinsic capacity to express higher levels of cytokines than peripheral blood NK cells (Cooper et al., 2001). Here, we used flow cytometry to analyze the expression of perforin, IFN-γ, IL-17A, TGF-β1, IL-4, IL-10, and the chemokines, IP-10 and IL-8. Perforin is a pore-forming cytolytic protein that is found in the granules of cytotoxic T lymphocytes (CTLs) and NK cells. IFN-γ and IL-17A are proinflammatory cytokines that mainly mediate the cellular immune response, while IL-4 and IL-10 are anti-inflammatory cytokines that mediate the humoral immune response. TGF-β1, a member of the TGF-β superfamily, could inhibit the differentiation of lymphocytes and the production of cytokines. The main role of chemokines is to control the migration of cells. The expressed levels of perforin (91.28 ± 1.33%), and TGF-β1 (44.10 ± 6.84%) in the SGA group were significantly higher than those in the AGA group (perforin: 66.48 ± 5.69%, *p* = 0.0099; TGF-β1: 33.17 ± 11.38%, *p* = 0.0055, respectively). There were no significant differences between the two groups with regards to the other cytokines (Figure 4).

### dNK Cells in the SGA Group Showed a Reduced Ability to Promote Trophoblasts Proliferation

**FIGURE 5:**
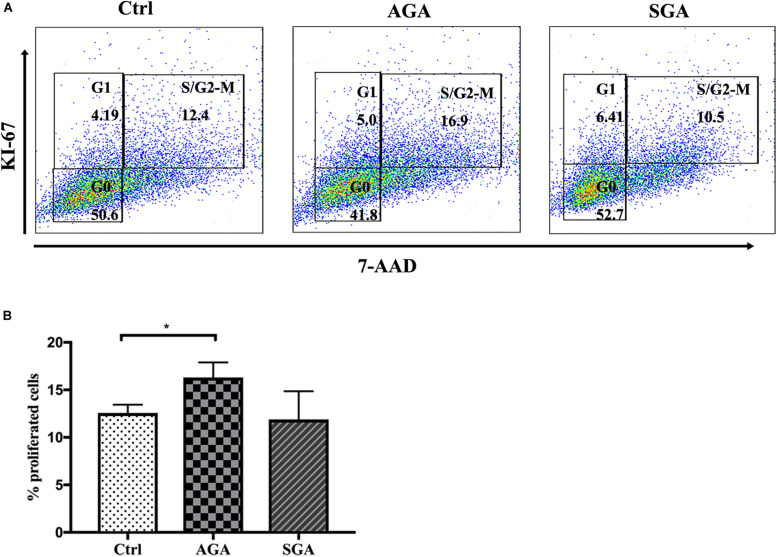
The effect of dNK cells on trophoblast proliferation. **(A)** Representative flow cytometry analysis of the expression of proliferating cells (S/G2-M) in complete medium (Ctrl), and when co-cultured with dNK cells in AGA (AGA), or SGA (SGA), respectively. **(B)** Statistical analysis of proliferating cells in the three different groups (*n* = 5). *p < 0.05.

Following implantation of the blastocyst into the endometrium, trophoblast cells continue to divide, proliferate, and differentiate to form villi. These villi further extend into the decidua basalis to form the chorion frondosum and, together with the decidua basalis, create the placenta. To investigate the effect of dNK cells on trophoblast proliferation, we directly co-cultured HTR8/SVneo and dNK cells from SGA and AGA for 24 h. The dNK cells were then discarded and the HTR8/SVneo cells collected for analysis. Flow cytometry was then used to determine the proportion of 7-AAD^+^Ki-67^+^ HTR8/SVneo cells; this population represented the cohort of S/G2-M proliferating cells. The proportion of proliferating cells co-cultured with dNK cells from the AGA group (16.3 ± 1.6%) was significantly higher than that in the control (HTR8/SVneo cells cultured without dNK cells) (12.58 ± 0.87%) (*p* = 0.0440), while there was no significant difference between the proportion of proliferating cells co-cultured with dNK cells from the SGA group and the control (Figure 5). These data demonstrated that dNK cells can promote the proliferation of trophoblasts in AGA, but the ability of dNK cells to cause such proliferation in SGA was far less obvious.

### dNK Cells in the SGA Group Exhibited a Reduced Ability to Promote the Migration and Invasion of Trophoblasts and HUVECs

**FIGURE 6:**
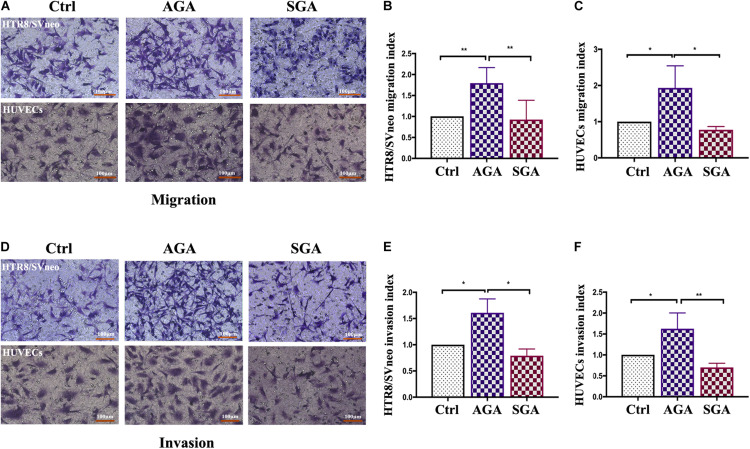
dNK cells in the SGA group attenuated the migration and invasion ability of trophoblasts and HUVECs. **(A,D)** Transwell assay results showing migrating **(A)**, or invading **(D)**, trophoblasts that reached the lower part of the filter membrane under the indicated conditions (Ctrl/AGA/SGA: with complete medium/with dNK from AGA/with dNK from SGA in the lower chamber, respectively). **(B,C,E,F)** Migration index **(B,C)**, or Invasion index **(E,F)** (number of migrated cells/the corresponding control). Statistical analysis was based on the results of six independently repeat experiments. *p < 0.05, **p < 0.005. Scale bars represent 100 μm.

Extra villous trophoblast cells (EVTs) invade the decidua, reconstruct spiral arteries and invade blood vessels, and maintain sufficient maternal blood flow to support placental function. Therefore, EVT invasion is an essential process for fetal implantation and placenta formation. We established a dNK/EVT and dNK/HUVECs co-culture system using a Transwell insert. HTR8/SVneo or HUVEC cells were seeded in the upper chamber of a Transwell insert with or without Matrigel, while isolated dNK cells were placed in the lower chamber. After 24 h of incubation, the migrated cells were photographed and counted. The numbers of migrated and invaded HTR8/SVneo or HUVEC cells were significantly higher in AGA than in the control group (HTR8/SVneo cells or HUVECs cultured without dNK cells) (*p* = 0.0022, *p* = 0.0239, respectively); however, there was no significant difference between the SGA and control groups. The numbers of migrated and invaded cells were significantly lower in the SGA group than in the AGA group (*p* = 0.0049, *p* = 0.0240, respectively) (Figure 6). This indicated that in AGA, dNK cells can promote the migration and invasion of trophoblasts or HUVECs, and that the ability of dNK cells to regulate the migration and invasion of trophoblasts or HUVECs was significantly weaker in the SGA group.

### dNK Cells in the SGA Group Exhibited Reduced Ability to Promote Tube Formation in Trophoblasts and HUVECs

**FIGURE 7:**
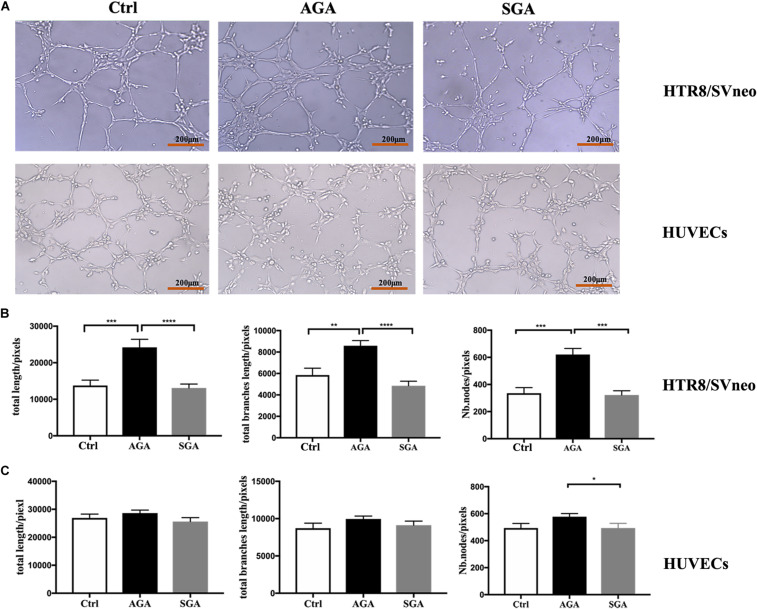
dNK cells in the SGA group exhibited reduced tube formation in trophoblasts. **(A)** Network-like structures formed by HTR8/SVneo cells and HUVECs in the condition of complete medium (Ctrl), pre-treated with dNK from AGA (AGA) or SGA (SGA). Scale bars represent 200 μm. **(B)** The total length, total branches length, and the number of nodes of HTR8/SVneo cells were calculated by ImageJ. **(C)** Total length, total branches length, and Nb. nodes of HUVECs were calculated by ImageJ. Statistical analysis was based on the results of six independently repeated experiments. **p < 0.005, ***p < 0.0005, ****p < 0.0001.

The remodeling of uterine spiral arteries is a vital process in placentation, which involves the participation of trophoblast cells and vascular endothelial cells. To investigate the effect of dNK cells on trophoblast cells and vascular endothelial cells, we first pre-treated HTR8/SVneo cells, or HUVECs, with dNK cells for 24 h. Then, the cells were added onto Matrigel and photographed 2 and 4 h later (Figure 7A). The total tube length, the total branch length, and the number of nodes in HTR8/SVneo cells from the AGA group were significantly greater than those in the SGA group and the control group (HTR8/SVneo cells or HUVECs cultured without dNK cells) (*p* < 0.0001 and *p* = 0.0003, *p* < 0.0001 and *p* = 0.0012, and *p* = 0.0001, and *p* = 0.0004, respectively). There was no significant difference between the SGA group and the control group (*p* = 0.7084, *p* = 0.1925 and *p* = 0.8004, respectively) (Figure 7B). However, the number of nodes in HUVECs in the SGA group was significantly lower than that in the AGA group (Figure 7C). This implied that in AGA, dNK cells have the ability to enhance the tube formation of trophoblasts but have no obvious effect on HUVECs; this effect was significantly reduced in SGA.

## Discussion

Small for gestational age infants are a major concern for pediatricians because these newborns not only have a high risk during the perinatal period but can also develop a series of problems during childhood and adulthood. Previous research studies have focused more on factors related to the occurrence of SGA (Catov et al., 2008; Palatnik et al., 2016; Englund-Ogge et al., 2018; Li et al., 2018; Manangama et al., 2019), postnatal management (Cardoso-Demartini et al., 2018; Visuthranukul et al., 2018), and health problems during growth (Gur et al., 2018). However, the exact pathogenesis of SGA remains unknown. A growing body of evidence suggests that SGA may be closely associated with impaired placental function, including placental vascular disease, pathogenic microbial infection, and abnormal changes in cytokine levels (Catov et al., 2008; Horgan et al., 2011; Lindner et al., 2013; den Hollander et al., 2017; Spiel et al., 2017). However, little is known about the immune microenvironment at the maternal–fetal interface of SGA infants.

The population of immune cells at the maternal–fetal interface predominantly features NK cells, CD3^+^ T lymphocytes, mononuclear macrophages, and dendritic cells. These cells, together with the local secretions of cytokines, chemokines, and extracellular matrix, constitute the immune microenvironment at the maternal–fetal interface. Maintaining a balance in this immune microenvironment is essential for normal pregnancy. Decidual NK cells are the key effector immune cells at the maternal–fetal interface in early pregnancy; they also monitor the invasion of trophoblast cells and maintain vascular modification and immune tolerance.

In this study, we collected decidua tissues from 24 cases of clinical SGA, along with decidua tissues from 48 cases of AGA as a control group. Our immunofluorescence assays showed that NK cells in the decidua maintained close contact with extra-villous trophoblasts. The percentage of NK cells in the decidua of SGA was significantly lower than that of AGA. Previous data, compiled by flow cytometry, indicate that the proportion of dNK cells is lower during the third trimester (30–40%) (Sindram-Trujillo et al., 2003) than during the first trimester (∼70%) (Bulmer et al., 2010; Fu and We, 2016). Our present findings confirmed that the proportion of dNK cells in the last trimester was 34.25 ± 2.30%, and that the proportion of dNK cells in SGA (25.01 ± 2.43%) was significantly lower than that in AGA. A similar decrease in the proportion of dNK cells was also observed in preeclampsia (PE) or fetal growth restriction (FGR) (Eide et al., 2005; Williams et al., 2009a). These pathological conditions will affect the growth and development of the fetus. Some overlaps in the pathogenesis of PE, FGR, and SGA might exist, but they would not be the same. We also found that the expression levels of the surface inhibitory receptor, NKG2A, on dNK cells in SGA were higher than that in AGA. These data demonstrated that the activity of dNK cells at the maternal–fetal interface was reduced in SGA. Cytotoxicity is an important functional indicator of NK cells; NK cytotoxic activity arises from an abnormal balance between inhibitory and activating signaling. In normal human early pregnancy, dNK cells mainly express the inhibitory receptor, NKG2A; this receptor binds to the non-classic MHC-I molecular HLA-E that is widely expressed by extra-villous cytotrophoblast. This interaction negatively regulates the cytolytic potential of dNK cells to maintain the immune tolerance of the fetus (Sotnikova et al., 2014). We further demonstrated that dNK cells in SGA exhibited lower cytotoxicity against K562 cells, and a greater ability to express TGF-β1 and perforin. TGF-β superfamily members are closely related to tissue remodeling and reproductive processes (Jones et al., 2006). dNK cells were reported to form conjugates and activate immune synapses with K562 cells; however, dNK cells failed to polarize their microtubule organizing centers and perforin-containing granules to the synapse, accounting for their lack of cytotoxicity (Kopcow et al., 2005). A decrease in cytotoxic potential in SGA is presented as a sign of the malfunctions of dNK cells. Together with the higher expression of NKG2A and TGF-β1, this may suggest that NK cells at the maternal–fetal interface may be relatively immunosuppressive in SGA. It is also a sign of increased tolerance of SGA. When compared to AGA, the proportion and function of dNK cells in SGA underwent significant changes, thus suggesting that changes in the immune microenvironment at the maternal–fetal interface may be related to the pathogenesis of SGA.

Trophoblast cells and vascular endothelial cells, which are important components of the placenta, have a significant impact on the growth and development of the fetus. Next, we attempted to investigate how dNK cells might influence placental trophoblast cells and vascular endothelial cells at the maternal–fetal interface in SGA. As described in previous research, we confirmed that in normal pregnancy, dNK cells can enhance the ability of the trophoblast cell line HTR8/SVneo or vascular endothelial cells (HUVECs) to proliferate, migrate, and invade. However, we performed these experiments with an identical number of dNK cells in SGA and found that these abilities were significantly weakened. This indicated that such effects are due to an abnormality in the function of dNK cells in SGA rather than a reduction in the number of dNK cells. Collectively, trophoblast migration and invasion provide the basis of placentation. Inadequate invasion of trophoblasts can lead to placental related diseases such as preeclampsia and intrauterine growth restriction and also affects overall fetal growth and development (Ridder et al., 2019; Duttaroy and Basak, 2020). During the first trimester of pregnancy, part of the cytotrophoblast will follow an invasive pathway and differentiate into an extra-villous trophoblast (EVT) (Judith and Cartwright, 2010; Tessier et al., 2015). Invasive EVT plays an active role in the remodeling of uterine spiral arteries (Tessier et al., 2015). The insufficient migration and invasion of trophoblasts will lead to poor placental formation and exert influence on fetal development. During human pregnancy, another type of trophoblast cell, referred to as endovascular extra-villous trophoblasts (enEVTs), penetrates the uterine spiral arteries and replace the endothelial cells (Smith et al., 2009; Ma et al., 2017; Moser et al., 2017; Sato, 2020). The uterine spiral arteries are then remodeled into utero-placental arteries, which provide the nutrition and oxygen that is necessary for fetal growth (Judith and Cartwright, 2010; Tessier et al., 2015). In the present study, we found that in AGA, dNK cells can significantly promote tube formation in HTR8/SVneo cells; however, this effect was not observed in the SGA group. This may suggest that the abnormal state of dNK cells may affect the function of trophoblast cells. Besides, immune dysregulation associated with elevated numbers of CD8^+^ cytotoxic T cells and mature dendritic cells were found during uterovascular remodeling in intrauterine growth restriction (IUGR) pregnancies (Dunk et al., 2019). This demonstrated that the process of vascular remodeling may be affected by the interaction of dNK cells and other decidual immune cells. Researchers also found that embryos derived from NK-depleted dams suffer from intrauterine growth restriction (IUGR) (Freitag et al., 2014); CD49a^+^Eomes^+^ subset of NK cells promoted fetal development through the secretion of growth-promoting factors in early pregnancies (Fu et al., 2017), these results suggested that dNK cells participated in fetal growth. The changes of dNK cells in SGA seem to be harmful to the function of placental trophoblasts and vascular endothelial cells, so we believe that they may also affect the growth and development of the fetus in another way.

Although our present results clarify the characteristics of dNK cells, and the possible mechanism of their effects on the growth and development of SGA fetuses, our research did have some limitations that need to be considered. Firstly, because the overall incidence of SGA is relatively low, it was difficult to source a sufficient number of SGA samples. Consequently, our work featured a relatively low sample size for the experimental group. In addition, it is worth mentioning that there are some differences between SGA and AGA in gestational age, which may affect some data. In future work, we will expand the sample size to compare SGA and AGA in the same gestational age, and investigate the functional characteristics of other related immune cells, as well as their interaction with NK cells in the immune microenvironment of the SGA maternal–fetal interface. Secondly, trophoblast cell line HTR8/SVneo was used and the co-culture experimental system was performed *in vitro*, which could not fully represent the real situation *in vivo*. The isolation and acquisition of primary trophoblast cells and appropriate animal experiments are necessary to be carried out in order to provide stronger evidence for our current research in the following work. Finally, there were not enough data on biochemical assessments during pregnancy and histological evaluations of placentas which may help to better interpret the results (Triunfo et al., 2016a,b).

In general, the proportion and functional capability of dNK cells at the maternal–fetal interface in SGA underwent significant changes. We also found that the ability of dNK cells in SGA to promote proliferation, migration, invasion, and tube formation in HTR8/SVneo cells or HUVECs was significantly weaker than in AGA. These changes may affect placental function and lead to disorders of fetal growth and development. Therefore, the results of this study strongly suggest that the abnormal functional capability of dNK cells in the immune microenvironment at the maternal–fetal interface is likely to be involved in the pathogenesis of SGA; the function of dNK cells is indeed an important factor for the growth and development of the fetus from the perspective of late gestation. Consequently, by regulating the functional status of the immune cells at the maternal–fetal interface, it might be possible to prevent and control the occurrence and development of SGA.

## Data Availability Statement

The datasets generated for this study are available on request to the corresponding authors.

## Ethics Statement

The studies involving human participants were reviewed and approved by Medical Ethics Committee of West China Second Hospital of Sichuan University. The patients/participants provided their written informed consent to participate in this study.

## Author Contributions

HL conceived and supervised the project and critically revised the manuscript. FL, CY, SY, and RZ helped collect the tissues and blood samples. FL, CY, TF, and SY performed the flow cytometry. FL, CY, and SY designed and performed most of the functional experiments. FL contributed to data analysis and wrote the manuscript. HL and RZ provided support and technical assistance. All authors read and approved the final version of the manuscript.

## Conflict of Interest

The authors declare that the research was conducted in the absence of any commercial or financial relationships that could be construed as a potential conflict of interest.
